# 

**Published:** 1858-03

**Authors:** 


### DENTAL REGISTER

### OF THE WEST.

EDITED BY

J. TAFT AND GEO. WATT.

**March, 1858.**

Published Quarterly at $3 Per Annum.

CINCINNATI:
WRIGHTSON & CO., PRINTERS.
1857.

